# Young Adults’ Perceptions on Life Prospects and Gender Roles as Important Factors to Influence Health Behaviour: A Qualitative Study from Karachi, Pakistan

**DOI:** 10.5539/gjhs.v4n3p87

**Published:** 2012-05-01

**Authors:** Syed Farid-ul-Hasnain, Eva Johansson, Ingrid Mogren, Gunilla Krantz

**Affiliations:** 1 Department of Community Health Sciences, Aga Khan University, Karachi, Pakistan; 2 Department of Public Health Sciences, Division of Global Health (IHCAR), Karolinska Institutet, Sweden; 3 Nordic School of Public Health, Gothenburg, Sweden; 4 Department of Clinical Science, Obstetrics and Gynaecology, Umea University, Umea, Sweden; 5 Department of Community Medicine and Public Health, The Sahlgrenska Academy at University of Gothenburg, Gothenburg, Sweden

**Keywords:** gender inequality, gender roles, young adults, health behaviour, HIV/AIDS

## Abstract

The purpose of this qualitative study was to explore perceptions and expectations of young males and females, in Karachi, Pakistan, regarding their life prospects and gender roles, with resulting implications for health behaviour. The main theme emerging was “Young adults’ prospects in life are hampered by psychosocial and gender equality constraints”. Gender inequality and the low status of women in society were described as major obstacles to the prosperity and development. Persistent withholding of information to the younger generation on sexual and reproductive health issues was perceived to increase exposure to health risks, particularly sexually transmitted infections (STIs). The present study reveals new discourses on equality among young adults, pointing towards an increasing, sound interaction between the sexes and aspirations for more gender equal relationships. The study further reveals serious misconceptions about HIV/AIDS. Such views and awareness among the younger generation constitutes a strong force towards change of traditional norms, including reproductive health behaviour, and calls for policy change.

## 1. Introduction

Growing into adulthood is shaped by social class and prevailing gender patterns and these experiences influence the life prospects of an individual (Furstenberg 2008; Sacker and Cable 2009). Rapid globalization, a process characterized by growing interdependence among people, has given the younger generations access to better communication means and has changed the pattern of interaction between the sexes. In a low income country like Pakistan, where western cultural influence is strong, such patterns also reshape the young adult’s identity (Arnett 2002). These changes have great implications for their demands of education and employment, and in redefining their gender roles, which eventually change their sexual and reproductive health behaviour.

Pakistan is foreseeing a large cohort of young people as those less than 15 years today comprise approximately 40% of the total population (Population Reference Bureau 2007). Adolescents in Pakistan live in an environment where lack of education and gender inequality are prevalent (Adolescents and Youth in Pakistan 2003). According to a United Nations Educational Scientific and Cultural Organization (UNESCO) report from 2006, the adult literacy rates in Pakistan is 62% for males and 38% for females (UNESCO France, 2005). Poor school enrolment and a high number of female dropouts, mainly among girls, further worsen the situation (Farid-ul-Hasnain and Krantz 2011). Young adults in Pakistan strive for better employment opportunities to secure their future. A report on labour force attendance revealed a decrease in employment rate for the youth from 42% in 2005 to 2006 to 40.9% in 2006 to 2007 (Ministry of Labour, Manpower and Overseas Pakistanis et al. 2008).

In Pakistan, a woman’s status is considered lower than that of her male partner, moreover male members of the family exert a strong influence on all women’s health related matters (Agha 1999). Women in professional settings face gender based resistance from the male dominant system (Mumtaz, Salway et al. 2003). A systematic review of the published literature reveals persistence of ‘honour killings’ in Pakistan, where homicidal acts are committed primarily against women (Patel and Gadit 2008). Gender inequalities in the form of son preference further underline the gender discrimination in the society (Hussain, Fikree et al. 2000). In the wake of the HIV/AIDS epidemic, gender inequality in the form of exploitation of women is one of the key factors in spreading HIV infection (Ali, Khanani et al. 2006). Poverty and gender inequalities are interwoven and potentiate each other in a vicious cycle. Financial constraints within the family often result in withholding girls’ education (Farid-ul-Hasnain and Krantz 2011). Girls are more likely to end up in early marriages and child-bearing, thus reducing their educational and economic opportunities, which will further enhance women’s poverty and low socio-economic status (Siddiqui 2007).

Cultural characteristics and ingrained behaviour play a role in maintaining gender inequalities. Women are in a particularly difficult situation with hardly any decision-making power within the family and extremely limited opportunities to decide about how to live their own life; whether to get married, whom to marry and how many children to give birth to. There is a need to address women’s health outcomes in the context of improving their right to freedom and equality of opportunities (Baptiste, Kapungu et al. 2010).

In our previous studies, we have presented evidence on the extremely low level of knowledge and awareness that exist among both boys and girls in relation to sexual and reproductive health and sexually transmitted infections (STIs) (Farid-ul-Hasnain, Johansson et al. 2009). In this paper, perceptions and expectations of young males and females (17-21 years) are addressed, regarding their future life prospects and gender roles in the metropolitan city of Karachi, Pakistan. It aims to catch some of the current attitudes that will influence health behaviour among men and women aged 17-21 years. Understanding this more in-depth has implications for policy and practice, for designing health promotion messages and health programs.

## 2. Methodology

A qualitative study design, using focus group discussions (FGDs) was adopted. In FGDs, participants actively discuss a specific topic in building new knowledge about social processes (Krueger RA 1994; Morgan DL 1997). In this study, we wanted to catch the interaction between the participants, their viewpoints, perceptions and ideas on prospects in life and their gender equality attitudes. However, as this is a sensitive topic, the participants were instructed not to reveal any personal experiences but to discuss in general terms what they believed was the current notion among people in the age group 17-21. Moreover, young people in Pakistan would hesitate to discuss such matters in individual interviews (Dahlgren L 2004).

Altogether, six FGDs were conducted, two from each socio-economic stratum, all being sex specific. The number of participants in each FGD varied from 6-8, with their age ranges from 17-21 years. Discussions were held at a venue according to the convenience of the participants, either at a nearby health centre, in a home or at a nearby community office and performed in the local language Urdu. The characteristics of the FGDs are presented in Table 1.

**Table 1:** Characteristics of the focus groups

FGD	Gender	No of Informants	Socio economic status (SES)	Venue
**G1**	Males	7	Low SES	Health centre
**G2**	Females	8	Low SES	Health centre
**G3**	Males	8	Lower middle SES	Participants’ home
**G4**	Females	6	Lower middle SES	Participants’ home
**G5**	Males	7	Upper middle SES	NGO office
**G6**	Females	6	Upper middle SES	NGO office

Informed consent was obtained from all the participants after briefing them on the study objectives and informing them that the discussions were to be tape recorded and how the confidentiality of the data would be maintained. The data were collected in December 2008.

### 2.1 Study Setting and Participants

This study was carried out in the metropolitan city of Karachi, Pakistan, which is the centre of industry and trade and comprises people from all areas of the country. In order to capture as many aspects and views of young adults as possible, the participants were chosen purposively (Khan, Anker et al. 1991), from three different segments of the city of Karachi. The inclusion criteria were single males and females of the age group 17-21 years, belonging to low, lower-middle and upper-middle socio-economic strata. The classification of socio-economic strata was made based on the place of residence. The characteristics of the participants are presented in Table 2.

**Table 2:** Socio-demographic characteristics of focus group participants

*Characteristics*	*N=42*
*Gender*	
Males	22
Females	20
*Age (in years)*	
17-18	18
19-21	24
*Educational attainment*	
None	3
Up to grade IV	1
Up to grade VIII	3
Up to grade IX	4
Up to grade X	3
Up to grade XI	8
Up to grade XII	5
Graduate studies	15
*Place of residence*	
Sultanabad (Low SES)	15
Qayyumabad (Lower middle SES)	14
Garden East (Upper middle SES)	13
*Occupational status*	
Non-working (including student)	32
Working	10

*Note: All the participants were unmarried*

The choice of age span, i.e. the youngest being those who had completed their secondary education ranging to those who were to enter into university or technical college, was to assess their views on the importance of education but also on gender issues for their future life as it will have an impact on their health behaviour. ‘Emerging adulthood’ is proposed as a new concept for the period from late teens through the twenties, which is the age for exploration and identity development (Arnett 2000).

Based on convenience different recruitment methods were used to identify participants. The first group belonged to lower socio-economic stratum *(Sultanabad)* and was identified with the assistance of a community coordinator working in the primary health care centre in the same area. The second group belonged to the lower middle socio-economic stratum *(Qayyumabad)*, and was identified with the help of the research assistant in this study, whereas the third group comprised people from the upper middle socio-economic stratum *(Garden East)* identified through a Non-Governmental organisation (NGO) working in the area. The participants were then approached and asked for participation by the research team members who had good local knowledge in the respective areas. No compensation or incentives were given to the participants.

### 2.2 Data Collection

The data collection team comprised a Pakistani medical doctor (SFH, first author), with a public health background, a Pakistani nurse educator (co-researcher) with experience in qualitative research, a Pakistani research coordinator with much experience of working in communities and two Pakistani research assistants having experience in qualitative research projects. Two Swedish global public health researchers (GK and EJ, co-authors) supervised the project in all its phases as did a Swedish specialist in sexual and reproductive health (IM, co-author). This composition of the research team contributed different perspectives and visions to the study.

A topic guide (Dahlgren L 2004) was used to guide the discussions covering two main areas: i) perceptions about the importance of education for prospects in life and possible constraints in achieving such goals; ii) perceptions about division of tasks between males and females and current gender roles in the society. The topic guide was initially pilot-tested and revised accordingly. The FGDs with males and females were facilitated by a male moderator (SFH) and a female moderator (co-researcher), respectively. Note taking and tape recording of the FGDs were undertaken by the research assistants. Each FGD lasted 70-90 minutes.

The tapes were transcribed verbatim in the local language (Urdu); notes were also used to authenticate the translation. An English translation of the text was made by the research coordinator who is well educated in both languages and has good experience of translations. A third person re-examined the translations to look for any omissions or misunderstandings. Finally, the first author (SFH) thoroughly looked through all the transcribed and translated text from the notes and recordings for verification.

### 2.3 Analysis

The transcripts from the FGDs were analysed using qualitative content analysis (Graneheim and Lundman 2004). We followed the model of analysis presented by Graneheim and Lundman (Graneheim and Lundman 2004) in that the ‘manifest content’, (visible, obvious components) and the ‘latent content’ (underlying meaning) of the text were analysed. The ‘manifest content’, that is, what the text says, is often presented as codes, sub-categories and categories, while themes are seen as expressions of the ‘latent content’ i.e. at the interpretative level (Graneheim and Lundman 2004).

As a first step, complete transcripts were read several times by the first author (SFH) in order to fully understand the views of the participants. ‘Meaning units’ (Baxter 1991) that mirrored statements in the transcript, were then identified by highlighting phrases in the transcripts. These were later merged into ‘condensed meaning units’ thereafter ‘codes’ were identified without loosing the context (Graneheim and Lundman 2004). Finally, three of the researchers, [SFH, EJ, GK] independently of each other reviewed the codes and similar codes were grouped into sub-categories and categories. After systematically analysing the commonalities, variations and disagreements of the three researchers, the latent content was identified and expressed as one theme and two sub-themes, which cut across all the categories (Polit D.F; Hungler 1999).

### 2.4 Ethical Considerations

Ethical approval for the study was given by The Aga Khan University Ethical Review Committee (AKU-ERC). Participants were also ensured about the confidentiality and were briefed that their participation in this study was voluntary and that they had full right to withdraw at any stage during the study. The consents were taken not only from the participants but also from their parents as suggested by the AKU-ERC.

## 3. Results

The findings of this study are summarised and expressed in one major theme (latent content) “Young adults’ prospects in life are hampered by psychosocial and gender equality constraints”, with two sub-themes “Emerging life prospects are counteracted by social and gender inequalities” and “Gender roles in transition”. Each sub-theme was built up by categories, sub-categories and codes (manifest content), which are further, described in Table 3.

**Table 3:** Examples of codes, sub-categories, categories, sub-themes and theme from the content analysis of focus group discussions of males and females about life prospects and gender roles

Theme	Sub-theme	Category	Sub-category	Codes
**Young adults’ prospects in life are hampered by psychosocial and gender equality constraints**	Emerging life prospects are counteracted by social and gender inequalities	Educational demands and imbalance	A strive for good education	Acquire good education. Education to be priority. Quality education. Girls should be educated. Educated mothers are better. Fight illiteracy

			Gender aspect and resources as main constraints to education	Non- affordability. Poor Govt. support. Corruption
				Family restrictions. Cultural constraints. Transportation. Marriages

		Scarce job opportunities	Quest for better jobs	Good job. Earn good money. Successful life. Competitive.

			Structural barriers to paid employment	Corruption. Lack of jobs. Less freedom for girls. Male dominated society. Increased population.

	Gender roles in transition	*young women’s perceptions on cultural barriers		Male dominance. Gender discrimination. Parental restrictions. Cultural barriers. Lower status for women.

		Changing interaction patterns		Better interaction. Better socialization. Influence of media. Changing attitudes. Western thoughts. Generation gap. Gender awareness. Health risks awareness

		Male tasks		Earn money. Support family. Look after children. Share HH tasks. Cook food.

		Attributes of husband	‘Good’ Husband	Earn money. Caring. Equal family rights. Respects wife. Female autonomy.

			‘Bad’ Husband	Irresponsible. Disrespect. Dependent on wife. Domestic violence. Addict.

		Female tasks		All HH tasks. Cooking. Earn money. Care for in- laws. Look after children.

		Attributes of wife	‘Good’ Wife	Look after house. Caring. Respects in laws. Sincere. Better understanding. Save money. Trustworthy.

			‘Bad’ Wife	Careless. Irresponsible. Disrespects in laws. Does not obey husband. Materialistic.

		Perceptions about HIV/AIDS		Social isolation. Women blamed more. Few heard of HIV/AIDS. Incurable disease. Misconceptions About HIV/AIDS. Rapid spread

* Category from female FGDs only

### 3.1 Emerging Life Prospects are Counteracted by Social and Gender Inequalities

The participants discussed their perceptions about life prospects and its constraints, in relation to ‘Educational demands and imbalance’ and ‘Scarce job opportunities’, as shown in the categories and sub-categories of this sub-theme.

#### 3.1.1 Educational Demands and Imbalance

Young adults, both men and women, were eager to acquire higher education and stressed that high quality education should be for everyone. Moreover, female participants from low SES group with no formal education further expressed that an educated mother can nurture her child in a better manner than an uneducated mother.

…“Nowadays, education has become very expensive and only rich people can afford to educate their children from good institutions whereas lower and middle class families cannot. Unfortunately, girls cannot complete their education as in our society; parents want their daughters to get married as soon as possible. Parents should understand the importance of education, and they should discuss matters related to the marriage with their daughters before making decisions”. (Female upper middle SES)

The demand for good education was discussed by all the participants, but some of the male participants from low SES strongly emphasized that education should be available for both men and women to create better life circumstances for families in the future.

Participants discussed that the major barriers in acquiring education were poor governmental support and cultural constraints. Some females mentioned family restrictions and marriage as one of the barriers to higher education.

… “I think education is most important. Once we get married we have to look after our husband and children and then it’s really difficult or rather impossible to get access to education”. (Female from low SES)

Female participants also discussed parental restrictions and lack of a proper transport system as one of the constraints. Some of the male participants, specifically from low SES groups, identified corruption in the educational system as a barrier to a fair system guaranteeing high quality education for all.

…“Our education system is not transparent. Rich people can influence and manipulate their exam papers through money and their personal contacts, and unfortunately the poor are the sufferers”. (Male from low SES)

#### 3.1.2 Scarce Job Opportunities

The participants were eager to express their goals in life and willing to develop their potential in order to have a prosperous future and a peaceful life. Furthermore, female participants discussed that the current economic crisis necessitates females to seek employment.

…” A woman should not only take care of her household but also work to earn money to tackle the economic crises”. (Female from lower middle SES)

Some participants described corruption, lack of respectable jobs and less mobility for women as the greatest obstacles to employment. Female participants also discussed that the female employees face additional problems due to few career paths, poor working environments and low wages.

Moreover, male participants from low SES expressed worries related to the growing population as a potential obstacle to employment.

### 3.2 Gender Roles in Transition

The participants described gender roles in transition with regard to ‘young women’s perceptions on cultural barriers’, ‘changing interaction patterns’, ‘male and female tasks allocation’ and attributes of a good or a bad ‘husband’ and ‘wife’ and ‘perceptions about HIV/AIDS’ as described in the categories and sub-categories of this sub-theme.

#### 3.2.1 Young Women’s Perceptions on Cultural Barriers

Informants from different socio-economic strata described that society is primarily male dominant with open discrimination of women in employment with lower wages; moreover they identified cultural barriers and parental restrictions as barriers to female autonomy in seeking education and paid jobs.

… “Parents should allow their children to breathe, small restrictions are fine but girls should feel independent”. (Female from upper middle SES)

Most of the females expressed that the males should acknowledge the female independence and respect them accordingly.

“Due to acknowledged discrimination, women have become desperate and intolerant. Men do not seem to respect the members of the opposite sex. We should work towards the elimination of this discrimination and create awareness that both sexes are equal and they should be respected in the same way”. (Female from upper middle SES)

#### 3.2.2 Changing Interaction Patterns

All informants agreed on that with globalisation and modernization, interaction between boys and girls has increased. Participants unanimously appreciated this changing pattern. Also discussed were the younger generation’s higher level of gender awareness and the existence of a clear generation gap.

… “As in the past years, boys and girls were more conservative and avoided talking to each other even at school or college. Nowadays, things have changed as boys and girls not only interact when at school but also in their leisure time”. (Male from low SES)

These changes were attributed to the effect of the media, including cable TV and magazines. The young people of today frequently use the internet for *‘chatting’* and cell phones for ‘*messaging’*.

…” We are under a lot of influence from the Western culture, which is the reason why male and female interaction has increased in recent years. Media is also responsible for these changes”. (Male from lower middle SES)

#### 3.2.3 Male Tasks and Attributes

The informants were well aware about the traditional division of tasks between males and females. However, some of them discussed future gender roles and mentioned some additional tasks for men to fulfil, which culturally are identified as female tasks.

…“If the wife is also working, then the husband should cooperate and assist his wife in household tasks e.g. washing dishes, making tea etc”. (Female from lower middle SES)

The participants, both males and females from different socio-economic strata, described that a good husband should be caring, able to earn money, and respect his wife. A good husband should also share equal rights with his wife and respect female autonomy.

…“A good husband is one who takes care of the rights of his wife, and also looks after children, household and his parents”. (Male from lower middle SES)

The informants unanimously expressed that a bad husband is one who is irresponsible and quarrelsome; moreover he disrespects his wife and believes in domestic violence.

#### 3.2.4 Female Tasks and Attributes

In general, participants described the female tasks as ingrained in accordance with the local culture such as cooking, cleaning and to look after children. However, both male and female participants stated that a woman should also earn money.

…“Now, in a current more demanding situation with the globalization and economic development I feel both husband and wife should earn money to sustain and lead a happy life. I mean that the wife should also be in paid employment”. (Male from upper middle SES)

There was unanimous agreement among the participants that a good wife should be caring, able to look after the house, respect her in-laws and be trustworthy. Moreover, a good wife should be economical in spending money and share a good level of understanding with her husband.

Male and female participants from high and low social strata expressed the attributes of a bad wife as careless, irresponsible and materialistic.

#### 3.2.5 Perceptions about HIV/AIDS

The participants also discussed that increased interaction between males and females and limited knowledge on reproductive health issues may increase health risks making the younger generation vulnerable to STIs.

…“I think we cannot stop the male and female interaction because it is natural. We should rather work towards educating our youth about the hazards and risks of physical relationships and the advantages of using condoms etc”. (Female from upper middle SES)

The FGDs revealed that there is poor awareness about HIV/AIDS and that many misconceptions about HIV/AIDS prevail among young adults. Although the participants were aware that HIV/AIDS is a deadly and incurable disease, girls also perceived HIV/AIDS as a hallmark of modernization.

… “Today media, movies and internet have resulted in increased interaction and friendship among girls and boys. They tend to ignore all limitations and boundaries and get inspired from the western society and due to this, sexually transmitted diseases are increasing”. (Female from upper middle SES)

The younger generation was not fully aware of the risks and consequences of getting HIV/AIDS. Those who were less educated and living in underprivileged areas were more ignorant than others.

Stigma related to HIV/AIDS was described both by male and female informants as a societal disgrace. Female informants also described that women testing positive for HIV were looked upon as inferior as compared to infected men, and they were also blamed more than the males.

… “Our society considers those who are suffering from HIV/AIDS as having a bad character, people will never sit, eat or have physical contact with them. The society will disgrace a woman more than a man because our culture is like that”. (Female from lower middle SES)

… “If a man or a woman is suffering from HIV/AIDS, I would think and treat such a man or a woman in the same way and avoid the infected person and never think of living with him/her.” (Male from low SES)

## 4. Discussion

Our findings point at emerging new discourses of equality among young adults in urban Pakistan, developing as a result of globalisation and through new communication tools with internet access, cell phones and media exposure. Old taboos related to the non-interaction between the sexes during up-bringing are questioned and a growing quest for improved gender equality seems at hand. The men as well as the women in this study stressed the importance of higher education and paid employment also for women, task sharing in the domestic sphere and shared responsibility in family matters.

This change in gender role attitudes were also reflected in the criticism of traditional beliefs about male and female behaviours, but in the discussions on male and female tasks and responsibilities, traditional gender norm thinking became apparent among both men and women. This contradictory observation is of no surprise but rather what is seen in all societies (Niaz and Hassan 2006; Gill and Stewart 2010). The road from attitudinal change to change of practice takes time and needs support from society at large in the form of gender equal laws and regulations, institutional change, open debate and change of cultural practices.

### 4.1 Aspiration for Education

The current economic recession has influenced the views of young males and females. Young women from lower social stratum pointed at several major constraints to continue their education; parental restrictions, lack of means but also to the discriminatory practices they face as women in the labour market. The men worried about availability of paid employment. These findings are consistent with the findings of another study conducted among young adults in Karachi (Farid-ul-Hasnain and Krantz 2011). However, education for women specifically has been pointed out as one of the most important factors for societal development, as it will contribute to improve women’s decision-making capacity in politics and in business (Lutz and Samir 2011). It will further reduce the number of children born, the overall standard of living will improve and general health it will bring better health to the entire family (Lutz and Samir 2011). However, unemployment and gender gaps in the labour market (Ministry of Labour, Manpower and Overseas Pakistanis et al. 2008) eventually force the younger generation to work in informal settings instead of completing their education (Khan, Hameed et al. 2007). The informants in our study were well aware of this, which is a step towards change.

### 4.2 Perception of Gender Roles and Sexual and Reproductive Health Behaviour

Considering the diversity of the Pakistani society, female empowerment to combat serious gender inequalities has different meanings for women belonging to different social strata. Men and women from all socio-economic strata however pointed at the lower status of women in this society, and described how women are deprived of decision making power and independence. This is also consistent with another qualitative study conducted on young women in slum areas of Pakistan, which showed submissive attitudes of young women as a sign of their subordinate position to their husbands (Hamid, Johansson et al. 2009). The masculinity norm, forming men’s higher status and privileges in society as compared to women’s, looks different in different cultures and countries and predicts how men and women interact with each other. A higher level of gender equality ensure better health status specifically for women but also for men, and forms an essential element of human rights principles (Belhadj and Toure 2008).

This study further point at the vulnerability related to sexual and reproductive health matters among young adults due to inadequate knowledge about safe sexual practices, STIs and specifically HIV/AIDS. HIV was considered a serious and deadly disease, however with serious misconceptions about routes of transmission. Young people have to rely on sources of information such as peers, media and internet which can be questioned in relation to accuracy and depth. Women from lower SES groups pointed at the stigma attached and how it affects infected women more than infected men, with women being blamed for transmission and seen as of inferior status in society.

As media now portrays a notion more inclined towards the western culture rather than towards Pakistani moral values, the notion was that young peoples’ health behaviour will change towards earlier sexual debut and unsafe sexual practices. This might in the near future result in early pregnancies and increased prevalence of STIs including HIV/AIDS.

### 4.3 Methodological Considerations

We believe that the choice of using FGDs for data collection was a feasible strategy as participants seemed confident to discuss such sensitive topics surrounded by peers rather than by being interviewed individually (Dahlgren L 2004). FGDs offered a shelter for the informants to express their own experiences in a generalised manner. Participants had interactive discussions among themselves to highlight the issues. This study did not enrol participants from the high SES as it was almost impossible to approach them as they live in a protected environment.

#### 4.3.1 Trustworthiness

In order to ensure trustworthiness, various steps were undertaken during the data collection and data analysis. For data collection, FGDs were conducted in local language (Urdu), separately for males and females, with varying age groups and socio-economic strata, with male and female moderator respectively, which ensured an open discussion of the phenomena under study (Graneheim and Lundman 2004). Findings were also tape-recorded to authenticate the notes. In order to ensure data quality, two of the FGDs were back translated by a third person to verify the consistency of the text. A brief meeting was held after each FGD amongst all the Pakistani team members to ensure the data quality and to discuss any emerging topic. The triangulation of researchers in analysing data and sharing the preliminary findings increased the credibility of the findings (Dahlgren L 2004). Furthermore, the findings were also shared with the participants and they approved of the findings, which also ensured the credibility of the study (Dahlgren L 2004). The quotations given in the study are intended to facilitate the reader’s evaluation of the creditability of results (Graneheim and Lundman 2004).

As with qualitative studies in general, it is not possible to generalize the findings (Khan, Anker et al. 1991). However, the selection of participants with both males and females of varying age groups from different parts of Karachi and from different socio-economic strata, make it probable that a reasonable variation in findings was detected. It is further discussed that carefully conducted and analysed FGDs may be transferable to other population groups with similar characteristics (Krueger 1994; Kitzinger 1995). In this study, we were quite surprised by the rather small differences in opinions found related to socio-economic status so it might be that the perceptions expressed here are widespread among young adults in urban Karachi. Dependability and conformability of the study was ensured by following a ‘decision trail’ by sharing the analysis plan and the citations with another researcher not involved in the study (Dahlgren L 2004). This person was asked to combine the selected citations with the sub-categories, categories and main theme. As there was congruence between researchers within and outside of the study, the procedure strengthens trustworthiness of the study.

## 5. Conclusions

Pakistan, a strong Muslim society, needs a well-educated younger generation, able to support and take care of themselves as well as safe-guarding their own health. The present study reveals new discourses on equality among young adults, pointing towards an increasing, sound interaction between the sexes and aspirations for more gender equal relationships. This study further reveals serious misconceptions about HIV/AIDS. Such views and awareness among the younger generation constitutes a strong force towards change of traditional norms, including reproductive health behaviour, and calls for policy change.

Access to education and improved gender equality, although undeniably linked, need to be addressed individually (Ali, Krantz et al. 2011). Availability and quality of education is a governmental responsibility. A common formal merit system across all educational institutions needs to be developed for men and women to be equally judged when seeking paid employment.

More equal gender relations and educational achievements will together influence the rules of social behaviour which will have a profound influence on sexual and reproductive health behaviour of both sexes (Adinma and Adinma 2011). Such a transition will result in new health related challenges, which necessitates a more open attitude in society to sexual and reproductive health matters. Young people should be equipped with proper knowledge in this area. Parental guidance, school related health education, health care services able to respond to young people’s needs but also a strong support from religious leaders are required. Policy makers, institutions and non-governmental organizations need to respond appropriately to such demands and challenges.
